# On-farm antimicrobial usage in commercial turkey production in the United States, 2013–2021

**DOI:** 10.3389/fvets.2023.1158943

**Published:** 2023-06-05

**Authors:** Randall S. Singer, Nora F. D. Schrag, Isabel Ricke, Michael D. Apley

**Affiliations:** ^1^Department of Veterinary and Biomedical Sciences, University of Minnesota, St. Paul, MN, United States; ^2^Mindwalk Consulting Group, LLC, Falcon Heights, MN, United States; ^3^Livestock Veterinary Resources, LLC, Manhattan, KS, United States; ^4^Department of Clinical Sciences, College of Veterinary Medicine, Kansas State University, Manhattan, KS, United States

**Keywords:** turkeys, antimicrobial use, antimicrobial stewardship, epidemiological monitoring, bacterial enteritis, clostridial dermatitis

## Abstract

A key component of antimicrobial stewardship is the ability to collect antimicrobial use data and ultimately use this information to ensure that administrations are necessary and effective. National antimicrobial sales data cannot help in this capacity because the data lack context, for example, details concerning target species and disease indication. The objective of this study was to continue the development of a system for collecting flock-level on-farm antimicrobial use data from the U.S. turkey industry and to have it be representative of national turkey production in the U.S. This study utilized a public-private partnership to enable collection and protection of sensitive flock-level data from an extremely large industry while releasing deidentified and aggregated information regarding the details of antimicrobial use on U.S. turkey farms over time. Participation was voluntary. Data were collected for the period 2013 through 2021 and are reported on a calendar year basis. Using production statistics from USDA:NASS as a denominator, the data supplied by participating companies represented approximately 67.3% of turkey production in the U.S. in 2013, approximately 69.1% in 2017, and approximately 71.4% in 2021. The data that were submitted for 2021 are based on approximately 149,000,192 turkeys slaughtered and 4,929,773,506 pounds liveweight produced. Detailed prescription records representing approximately 60–70% of the birds were available for the 2018–2021 dataset. The estimated percentage of turkey poults placed that received hatchery antimicrobials decreased from 96.9% in 2013 to 40.5% in 2021. The use of in-feed antimicrobials was practically eliminated, with in-feed tetracycline being the only medically important antimicrobial used in 2021. Use of in-feed tetracyclines decreased approximately 80% between 2013 and 2021. Water-soluble antimicrobial use declined over the study period. Between 2013 and 2021, water-soluble penicillin use decreased approximately 41% but water-soluble tetracycline use increased approximately 22%. Key diseases that were treated with water-soluble antimicrobials included bacterial poult enteritis and clostridial dermatitis. Efforts to reduce the incidence of these diseases would reduce the need for antimicrobial therapy, thereby enabling continued decreases in antimicrobial use without sacrificing animal welfare. However, this will require an investment in research to find efficacious and cost-effective mitigation strategies.

## 1. Introduction

Improving antimicrobial stewardship (AMS) in human and animal health is essential for maintaining the effectiveness of available antimicrobials (1–3). The American Veterinary Medical Association (AVMA) states that AMS involves “maintaining animal health and welfare by implementing a variety of preventive and management strategies to prevent common diseases; using an evidence-based approach in making decisions to use antimicrobial drugs; and then using antimicrobials judiciously, sparingly, and with continual evaluation of the outcomes of therapy, respecting the client’s available resources” (3). To accomplish these goals in animal agriculture, systems must be developed for collecting on-farm antimicrobial use (AMU) data. These data should include the principal indications for use and details about administration (dose, route, duration, age of animals) of specific antimicrobial compounds, and ideally, would also collect information on therapeutic outcomes (2).

Most AMU data that are being collected globally are in the form of antimicrobial sales volumes; most countries do not currently have systems in place to collect nationally representative AMU datasets on-farm. Antimicrobial sales data represent the overall volume of antimicrobials sold or distributed through various outlets by the drug manufacturer and not the amounts actually used. They provide no information regarding the intended reason for use and typically have no information about route or duration of administration. Further, target species are often estimates or best guesses. In a recent publication discussing how antimicrobial use data collected on-farm can help guide stewardship, the authors state that the antimicrobial sales data collected from 31 countries and reported by the European Surveillance of Veterinary Antimicrobial Consumption (ESVAC) (4) have helped with “AMS at the national level, such as setting targets for reducing overall sales” (5). Without any knowledge of the incidence of disease in specific herds or flocks nor the intended use of the antimicrobials included in the sales data, it is not clear how national antimicrobial sales data alone are useful in assisting with AMS activities. Setting antimicrobial reduction targets based on sales data ignores changes in animal populations as well as the ever-changing incidence of disease that might necessitate antimicrobial therapy, which varies geographically, seasonally, and annually.

As we have described previously (6), the U.S. Food and Drug Administration (FDA) has made key changes to antimicrobial policy to improve on-farm AMS. These changes include limiting the use of medically important antimicrobials in food-producing animals to necessary cases for animal health and requiring veterinary oversight for administering these antimicrobials in feed or water. The FDA’s Guidance for Industry #152 defines “medically important” antimicrobials and provides a list and ranking of these antimicrobials considered important in the US, which serves as a classification system for data presented in this US-based effort. One change was the requirement of veterinary oversight for medically important antimicrobials administered in the feed or water of food-producing animals, either as a Veterinary Feed Directive (VFD) order (for feed-administered medically important antimicrobials) or prescription (for medically important antimicrobials administered in water) (7–9). The U.S. has its own list of antimicrobials considered medically important to human medicine, which can be found in FDA’s Guidance for Industry (GFI) #152 (10); in this study, we use the FDA list as the system for classifying the data presented in this U.S.-based effort.

Although the U.S. FDA collects and reports national antimicrobial sales data on annual basis (11), these data cannot replace AMU data collected on-farm. In 2020, we published data regarding on-farm use of antimicrobials in U.S. turkey production (6). This national effort represented more than 70% of the annual turkey production in the U.S, with more than 160,000,000 slaughtered turkeys and more than 5 billion pounds liveweight represented in the 2017 data. That effort covered the period 2013–2017 and categorized the use of antimicrobials on-farm by route of administration (hatchery, feed and water). We also reported the disease indications for the 2017 water-soluble antimicrobials. This new data collection effort, which covers the period 2018–2021, aimed to provide more information on the diseases being treated, the age of onset at the time of treatment, the duration of therapy, and the number of prescriptions each year. The goal was to have the data collected be representative of the national turkey flock. Some companies updated their records in the 2013–2017 dataset to be consistent with the format of the 2018–2021 dataset.

## 2. Materials and methods

### 2.1. Enrollment

Our overall aim was to enroll the companies that raise the majority of commercial turkeys in the U.S. Data from the USDA National Agricultural Statistics Service (NASS) report that approximately 213,937,00 turkeys were slaughtered in 2021 (12). We have previously published details regarding enrollment for this project (6). In this second phase of the project, flock-level treatment records were requested from participating companies, particularly for the data from 2018 through 2021. Participation was voluntary, and all companies were guaranteed data confidentiality and that only industrywide aggregated data would be released publicly.

### 2.2. Data collection, aggregation, and reporting

Details about the data collection process are presented in our previous publication (6). Briefly, all collected data were aggregated in yearly totals. Companies all had different formats for recording and reporting data, and consequently, manual curation of the company datasets was necessary. Many of the companies were able to provide the data as flock-level prescription records. However, some data, particularly data related to the in-feed use of antimicrobials, were submitted as calendar year totals. Data provided as calendar year totals are similar to antimicrobial sales data because they do not have the details of AMU administration. However, unlike national antimicrobial sales datasets, the disease indication estimates for these data provided some context for the actual AMU.

Our previous publication provides information regarding data validation and aggregation (6). After validation, data from each company were imported into R 4.2.2 (13) and aggregated. All analyses and graphing of the AMU records were performed in R.

Antimicrobial use estimates are stratified into medically important (MI) and not medically important (NMI), as defined by FDA (10), and details regarding these classifications are shown in Supplementary Tables S1 and S2. Within each classification of medical importance, we present the AMU data by active substance within each class. Due to differences in dose and potency/molecular weight of the antimicrobial substances reported in this project, no attempts were made to combine the AMU data across antimicrobial classes or routes of administration (11).

For AMU totals, we report the estimates in a similar fashion to our previous publication (6). Briefly, antimicrobials used in the hatchery are reported as totals and as mg per 100 poults placed. The in-feed and water-soluble administration data are reported as totals and as mg/kg liveweight slaughtered. All AMU totals that were collected during this study are included in the Supplementary material.

### 2.3. Granular antimicrobial use data analysis

For the 2018 through 2021 data, many of the participating companies submitted flock-level treatment records for antimicrobials administered *via* the water. The data that had this level of granularity represented between 60 and 70% of the turkeys in the 2018–2021 dataset. The full prescription records included the disease being treated, the number of animals being treated, the age of the birds at the start of therapy, the duration of therapy, and the amount of antimicrobial sent to the farm for the prescription. Not all records had all of this information; for example, age at time of therapy was not always included. Throughout this study, we refer to these as prescriptions and not treatments because the records that we received were the actual prescriptions written by the veterinarian and were not records of actual on-farm treatment. While likely a rare occurrence, there is the possibility that the prescribed antimicrobials were never actually given to the animals, and thus prescription is more accurate than treatment.

With these more granular records, several analyses were conducted, and only those records that included the specific information described below were included in the respective analysis. These analyses are stratified by disease indication, and not all veterinarians or companies use the same terminology for each disease. Veterinarians were contacted to clarify some of the reported disease indications and how best to aggregate the prescriptions into broad categories. First, the number of water-soluble prescriptions written for each disease indication was estimated by year. Second, the distribution of ages at the start of therapy for the most commonly treated disease indications was calculated, represented by the number of birds beginning treatment for each disease indication by week of age. This analysis was conducted on data collapsed over the 2018–2021 period. Third, the number of prescriptions of each antimicrobial class was stratified by disease and year. Fourth, the distributions of prescription durations for each disease indication were calculated using data collapsed over the 2018–2021 period. Finally, the percentage of each water-soluble antimicrobial that was administered for each disease indication was estimated by calculating the percentage of birds treated and the total grams of each antimicrobial administered for each disease indication.

## 3. Results

### 3.1. Enrollment

The data that were submitted for this phase of the project represented the majority of turkeys produced in the U.S. during each year of the study. All types of turkey production were represented in the dataset, including conventional, raised without antimicrobials (RWA) and organic production. Most of the companies that participated in the study produced animals in more than one of these production classifications, and consequently, the submitted data cannot be stratified by production type.

The 2013 dataset included 181,856,809 poults placed, 158,993,743 turkeys slaughtered and 4,857,483,649 pounds (lbs) liveweight produced in 2013 (Table 1). Participation increased between 2013 and 2017, and the 2017 dataset included 185,536,089 poults placed, 160,644,707 turkeys slaughtered and 5,125,329,005 lbs. liveweight produced (Table 1). The 2021 dataset included approximately 169,901,720 poults placed, 149,000,192 turkeys slaughtered and 4,929,773,506 lbs. liveweight produced (Table 1). As a percentage of turkeys slaughtered and total pounds liveweight produced in the U.S. in 2013, the dataset represented approximately 66.8 and 67.3%, respectively. These figures increased to approximately 69.6% and 71.4% in 2021. Participation rates were fairly constant over the 9 years of data collection. The Supplementary material contains all of the denominator data collected during the study.

**Table 1:** Turkey production data included in the antimicrobial datasets submitted by participating companies for each year of the study.

	2013	2014	2015	2016	2017
Hatchery antimicrobial denominators					
Poults Placed	181,856,809	183,534,540	182,914,240	192,829,005	185,536,089
Study Production Denominators					
Head Slaughtered	158,993,743	162,024,651	157,148,219	167,784,291	160,644,707
Liveweight (lbs)	4,857,483,649	4,987,227,055	4,783,181,828	5,250,562,956	5,125,329,005
USDA:NASS statistics					
Head Slaughtered	237,964,000	235,189,000	230,812,000	241,418,000	240,014,000
Liveweight(lbs)	7,220,540,000	7,150,782,000	6,972,127,000	7,412,058,000	7,420,122,000
Percentage of U.S. turkey production					
Head Slaughtered	66.8%	68.9%	68.1%	69.5%	66.9%
Liveweight(lbs)	67.3%	69.7%	68.6%	70.8%	69.1%

	2018	2019	2020	2021
Hatchery antimicrobial denominators				
Poults Placed	183,554,723	181,124,754	177,603,898	169,901,720
Study production denominators				
Head Slaughtered	156,174,309	155,719,652	155,785,544	149,000,192
Liveweight (lbs)	5,041,484,986	5,117,188,844	5,095,630,402	4,929,773,506
USDA:NASS statistics				
Head Slaughtered	235,198,000	225,915,000	221,323,000	213,937,000
Liveweight (lbs)	7,310,114,000	7,236,359,000	7,141,912,000	6,908,616,000
Percentage of U.S. turkey production				
Head Slaughtered	66.4%	68.9%	70.4%	69.6%
Liveweight (lbs)	69.0%	70.7%	71.3%	71.4%

Annual U.S. turkey production statistics are taken from USDA:NASS (12). The percentage of annual U.S. turkey production calculation is based on the study production data for the in-feed antimicrobials (numerator) and USDA:NASS data (denominator). The water-soluble antimicrobial data represented fewer birds in 2013–2015 (see Supplementary material).

### 3.2. Hatchery antimicrobials

Antimicrobials used at the turkey hatchery antimicrobials are generally given as a subcutaneous injection in the day-old poult. Gentamicin was the primary antimicrobial used in the hatchery. Although certain extra label uses of cephalosporins in livestock and poultry were prohibited by the FDA in 2012 (14), ceftiofur sodium used in the day-old poults is a labeled administration. Finally, there was some use of penicillin between 2013 and 2021. When used, penicillin was always administered in combination with gentamicin, typically as a half dose of each antimicrobial.

**Figure 1:**
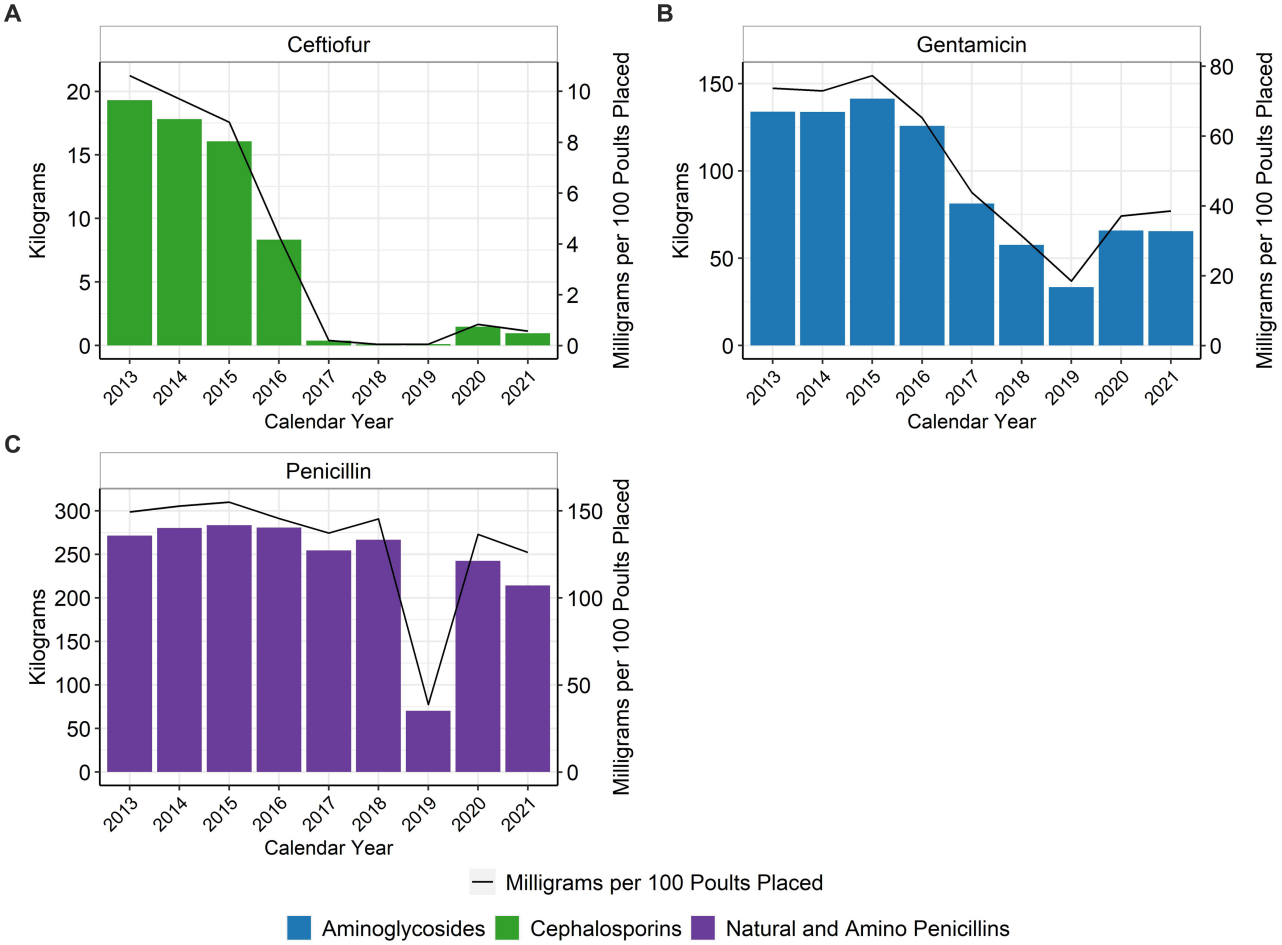
Ceftiofur **(A)**, gentamicin **(B)**, and penicillin **(C)** used in turkey hatcheries, 2013–2021. Total kilograms are shown by the bars (left Y-axis) and total mg/100 birds placed are shown by the line (right Y-axis).

**Figure 2:**
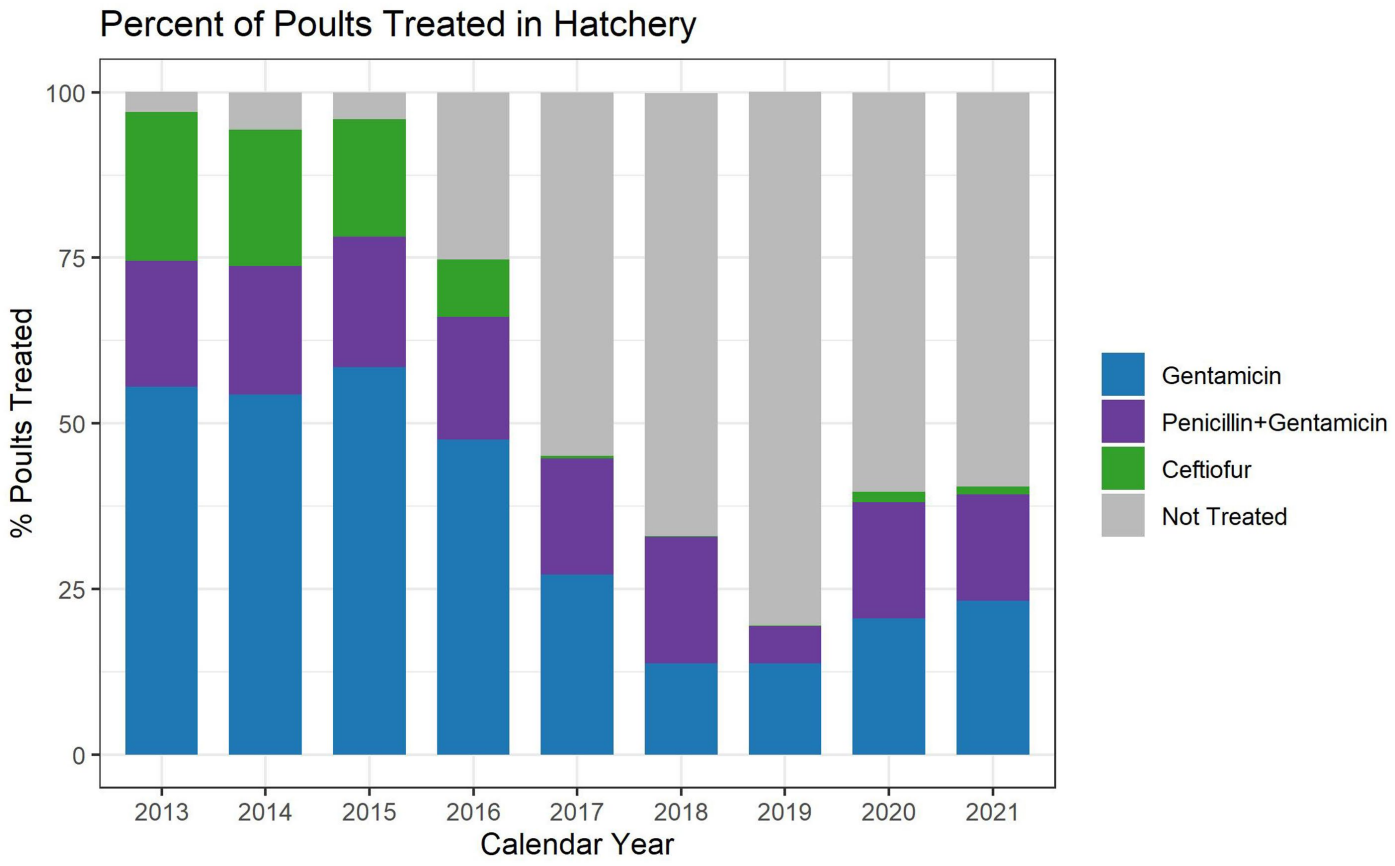
Turkey hatchery antimicrobial use during the years 2013–2021, as a percentage of total birds placed. The graph shows the percentage of birds placed that received gentamicin, ceftiofur, gentamicin and penicillin, or no antimicrobial.

The total number of turkey poults placed during study period ranged between 169,901,720 and 192,829,005, depending on the year (Table 1). In addition to total amounts of antimicrobial used, estimates of hatchery antimicrobial administration are also reported as total mg of active substance per 100 poults placed. Ceftiofur use decreased considerably over the study period, with lows in 2018 and 2019 (Figure 1A). Gentamicin use in the hatchery decreased approximately 47.6% between 2013 and 2021, with a low in 2019 (Figure 1B). Hatchery penicillin use declined approximately 15.6% between 2013 and 2021 (Figure 1C). The percentage of poults placed that received hatchery antimicrobials decreased from approximately 96.9% in 2013 to 40.5% in 2021, with a low of 19.4% in 2019 (Figure 2).

### 3.3. In-feed antimicrobials

**Figure 3:**
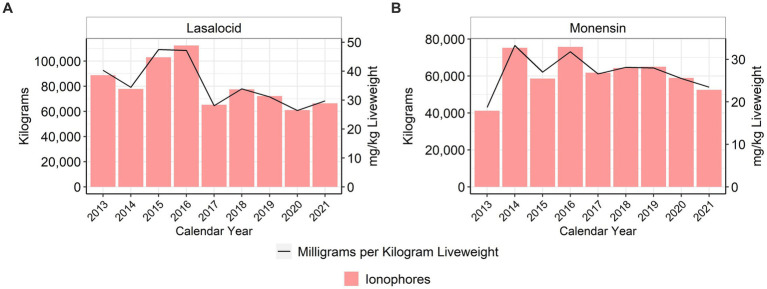
Lasalocid **(A)** and monensin **(B)** used in turkey feed, 2013–2021. Total kilograms are shown by the bars (left Y-axis) and total mg/kg liveweight are shown by the line (right Y-axis).

The ionophores lasalocid and monensin are approved for use in turkeys in the U.S. (15), where they are considered NMI antimicrobials, compared to their categorization as coccidiostats in many countries (4). When using the metric of total mg/kg liveweight produced, there was an approximate 26% reduction of lasalocid use between 2013 and 2021; monensin use increased approximately 25.6% between 2013 and 2021 (Figure 3). There were annual fluctuations in the use of the ionophores (Figure 3).

**Figure 4:**
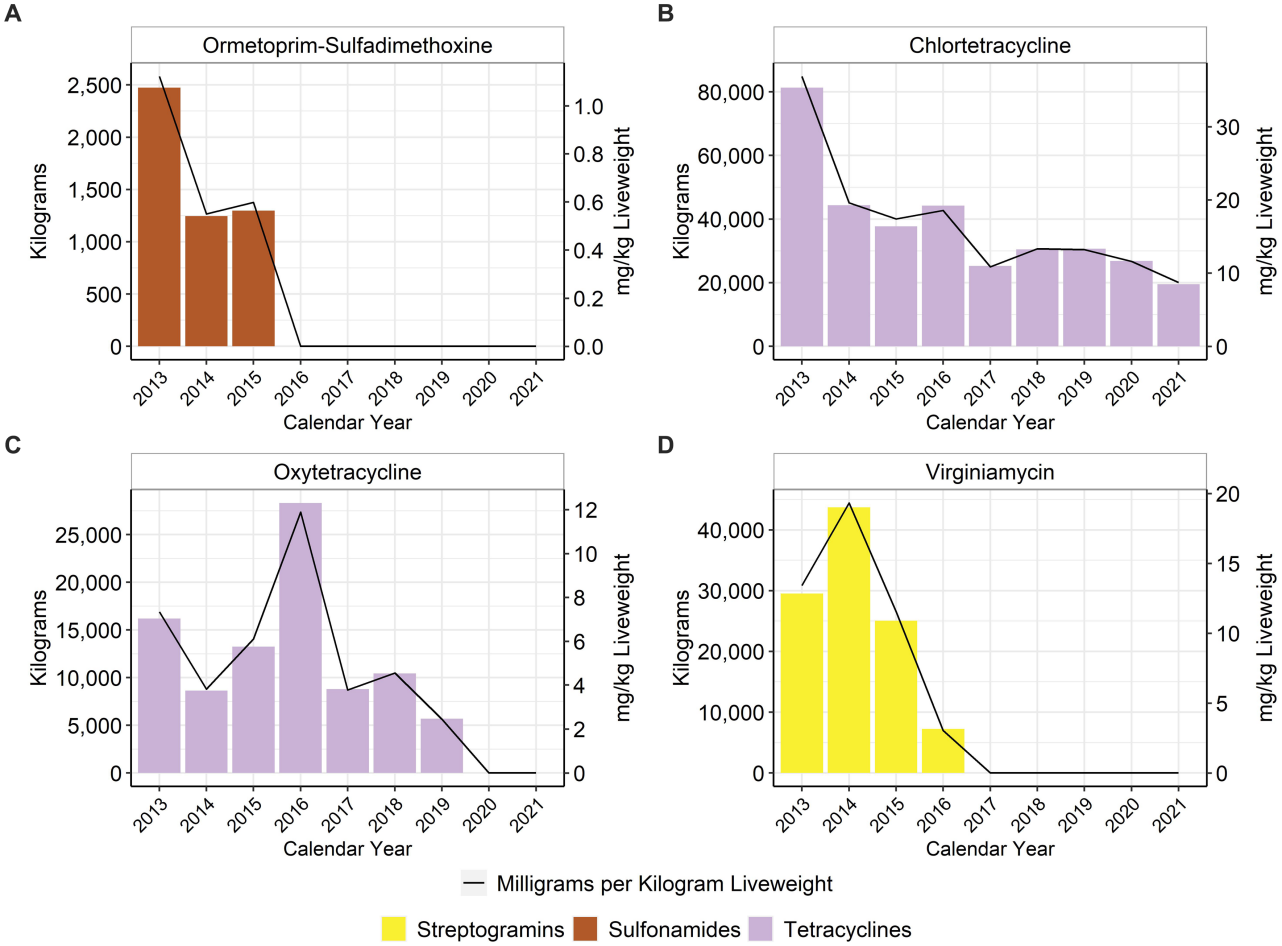
Medically Important antimicrobials ormetoprim- sulfadimethoxine **(A)**, chlortetracycline **(B)**, oxytetracycline **(C)**, and virginiamycin **(D)** used in turkey feed, 2013–2021. Total kilograms are shown by the bars (left Y-axis) and total mg/kg liveweight are shown by the line (right Y-axis).

**Figure 5:**
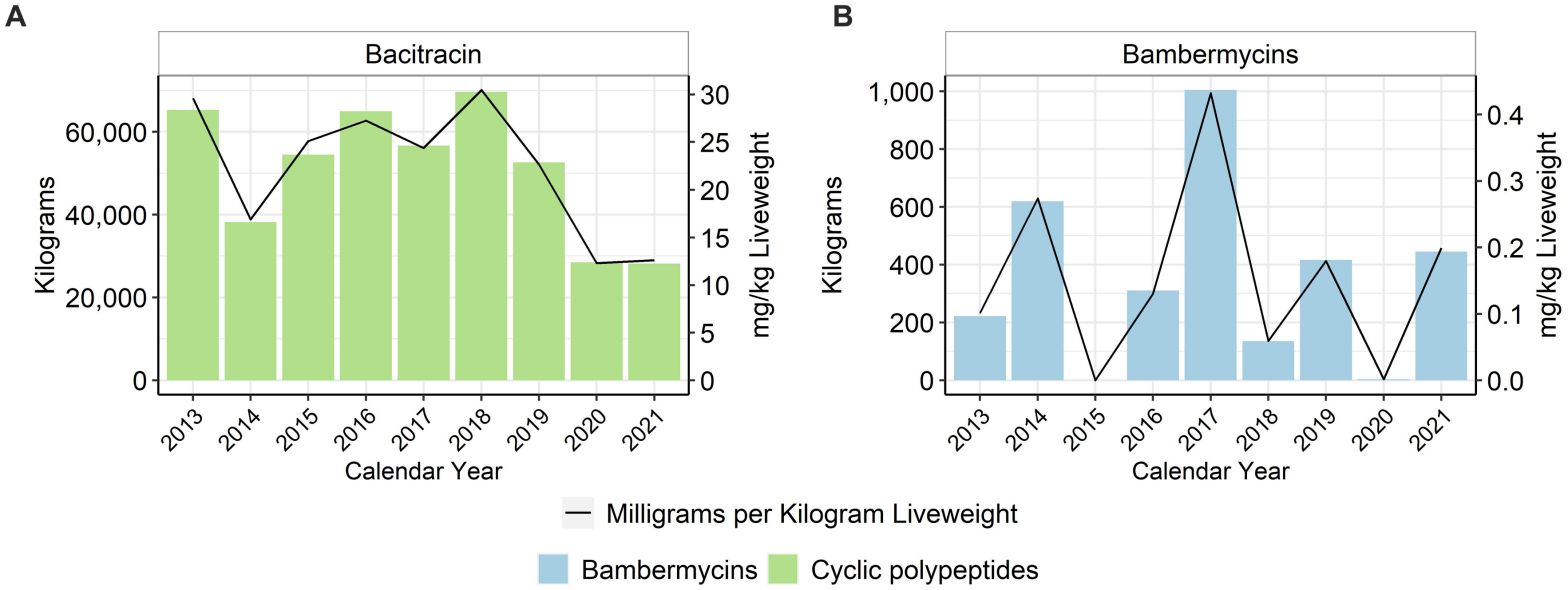
Not Medically Important antimicrobials bacitracin **(A)** and bambermycins **(B)** used in turkey feed, 2013–2021. Total kilograms are shown by the bars (left Y-axis) and total mg/kg liveweight are shown by the line (right Y-axis).

When using the metric of total mg/kg liveweight produced, there was an approximate 80% reduction in the use of tetracyclines (class) between 2013 and 2021 (Figure 4), with no oxytetracycline use reported in 2020 or 2021 (Figure 4C). Virginiamycin has not been used in the feed since 2016 due to the voluntary withdrawal of in-feed virginiamycin products that had previously included production claims such as increased feed efficiency for turkeys (Figure 4D). There has been no reported use of the combination antimicrobial sulfadimethoxine-ormetoprim since 2015 (Figure 4A). In-feed bacitracin, an NMI antimicrobial, decreased by approximately 57% between 2013 and 2021 (Figure 5A). Bambermycins, an NMI antimicrobial class labeled for production purposes, increased in usage between 2013 and 2021, with considerable variability year to year (Figure 5B).

### 3.4. Water-soluble antimicrobials

Water-soluble AMU data are reported for each active substance except for the sulfonamide class, where it can be challenging to separate the multiple water-soluble active substances from company records (Supplementary Table S1). There was reported water-soluble use of chlortetracycline, tetracycline and oxytetracycline, although oxytetracycline was used more frequently than the other two. Water-soluble administration remains the key manner for treatment of disease, as sick birds may consume less feed but will typically maintain water consumption (16).

In the U.S., there are fewer water-soluble antimicrobials approved for treatment and control of disease when compared to approvals for broiler chickens or for other non-poultry animal species. Consequently, extralabel use of water-soluble antimicrobials is often required (6, 17). Examples of drugs that were used in an extralabel manner in turkeys during this study period included lincomycin, trimethoprim-sulfadiazine, florfenicol and tiamulin.

**Figure 6:**
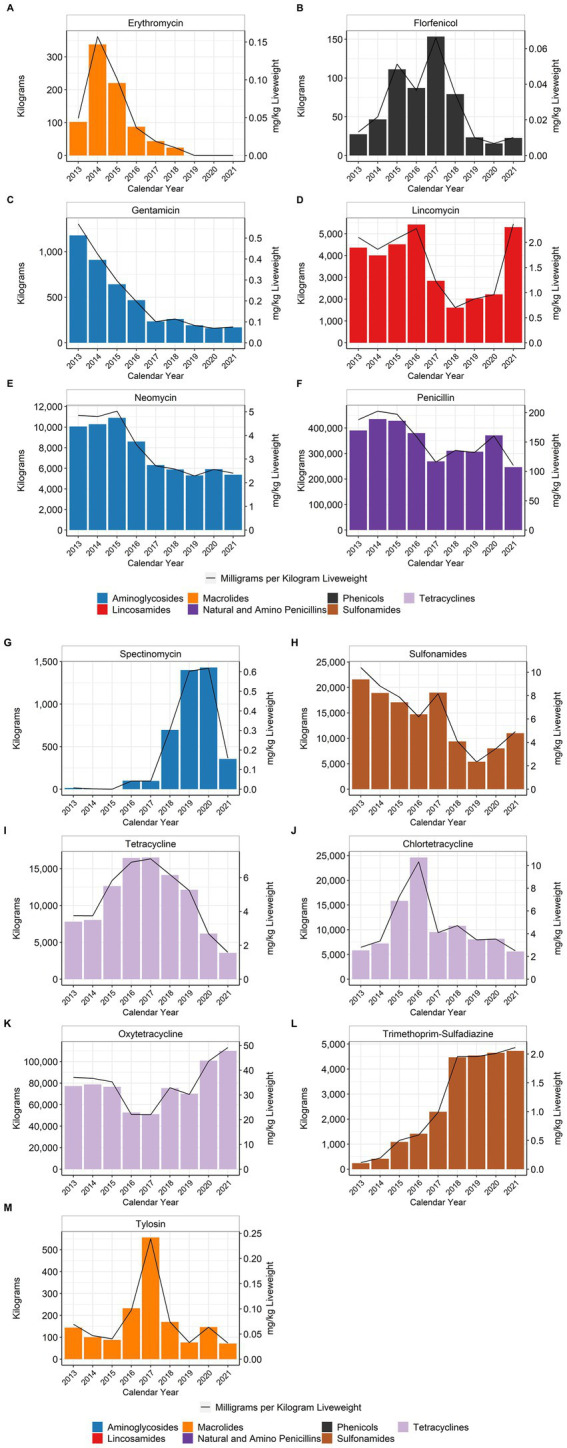
Medically Important antimicrobials erythromycin **(A)**, florfenicol **(B)**, gentamicin **(C)**, lincomycin **(D)**, neomycin **(E)**, penicillin **(F)**, spectinomycin **(G)**, sulfonamides **(H)**, tetracycline **(I)**, chlortetracycline **(J)**, oxytetracycline **(K)**, trimethoprim-sulfadiazine **(L)**, and tylosin **(M)** used in turkey water, 2013–2021. Total kilograms are shown by the bars (left Y-axis) and total mg/kg liveweight are shown by the line (right Y-axis).

**Figure 7:**
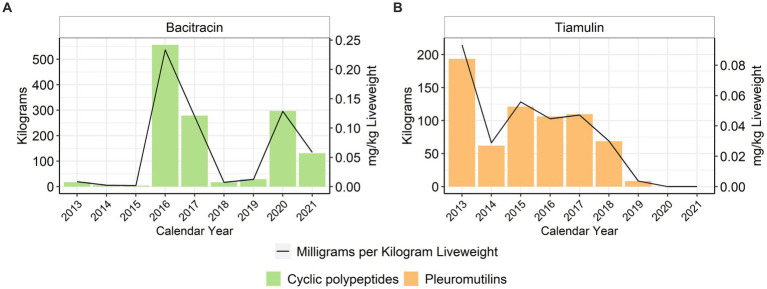
Not Medically Important bacitracin **(A)** and tiamulin **(B)** used in turkey water, 2013–2021. Total kilograms are shown by the bars (left Y-axis) and total mg/kg liveweight are shown by the line (right Y-axis).

When using the metric of total mg/kg liveweight produced, water-soluble penicillin use decreased approximately 41% between 2013 and 2021 with variability year to year (Figure 6F). Water-soluble lincomycin use decreased approximately 54% between 2013 and 2020, but then increased 147% from 2020 to 2021 (Figure 6D). Water-soluble tetracycline use, as a class, increased approximately 22% between 2013 and 2021 with variability year to year (Figures 6I–K). Water-soluble gentamicin use decreased approximately 87% from 2013 to 2021 (Figure 6C), while water-soluble neomycin use decreased approximately 50% during the same period (Figure 6E). Water-soluble erythromycin use decreased approximately 79% from 2013 to 2018; there was no reported use of erythromycin from 2019 to 2021 (Figure 6A). Trimethoprim is used as a combination drug with sulfadiazine; the formulation for the potentiated sulfonamide is 333 mg sulfadiazine and 67 mg of trimethoprim per mL. The trimethoprim-sulfadiazine grams are reported separately, but the sulfadiazine totals are also included with the general sulfonamide class totals (Figures 6H,L). Use of this compound formulation increased between 2013 and 2018 and then has remained steady through 2021 (Figure 6L). Tylosin, which is not permitted for in-feed use in turkeys, had an almost fourfold increase in its water-soluble use between 2013 and 2017 and then declined again (Figure 6M). The use of florfenicol increased almost fivefold from 2013 to 2017 and then declined again (Figure 6B), while spectinomycin use increased over the study period (Figure 6G). For the NMI antimicrobials, bacitracin and tiamulin were both used during the study period with varying patterns of use; there was no reported tiamulin use in 2020 or 2021 (Figure 7).

### 3.5. Granular antimicrobial use data analysis

**Figure 8:**
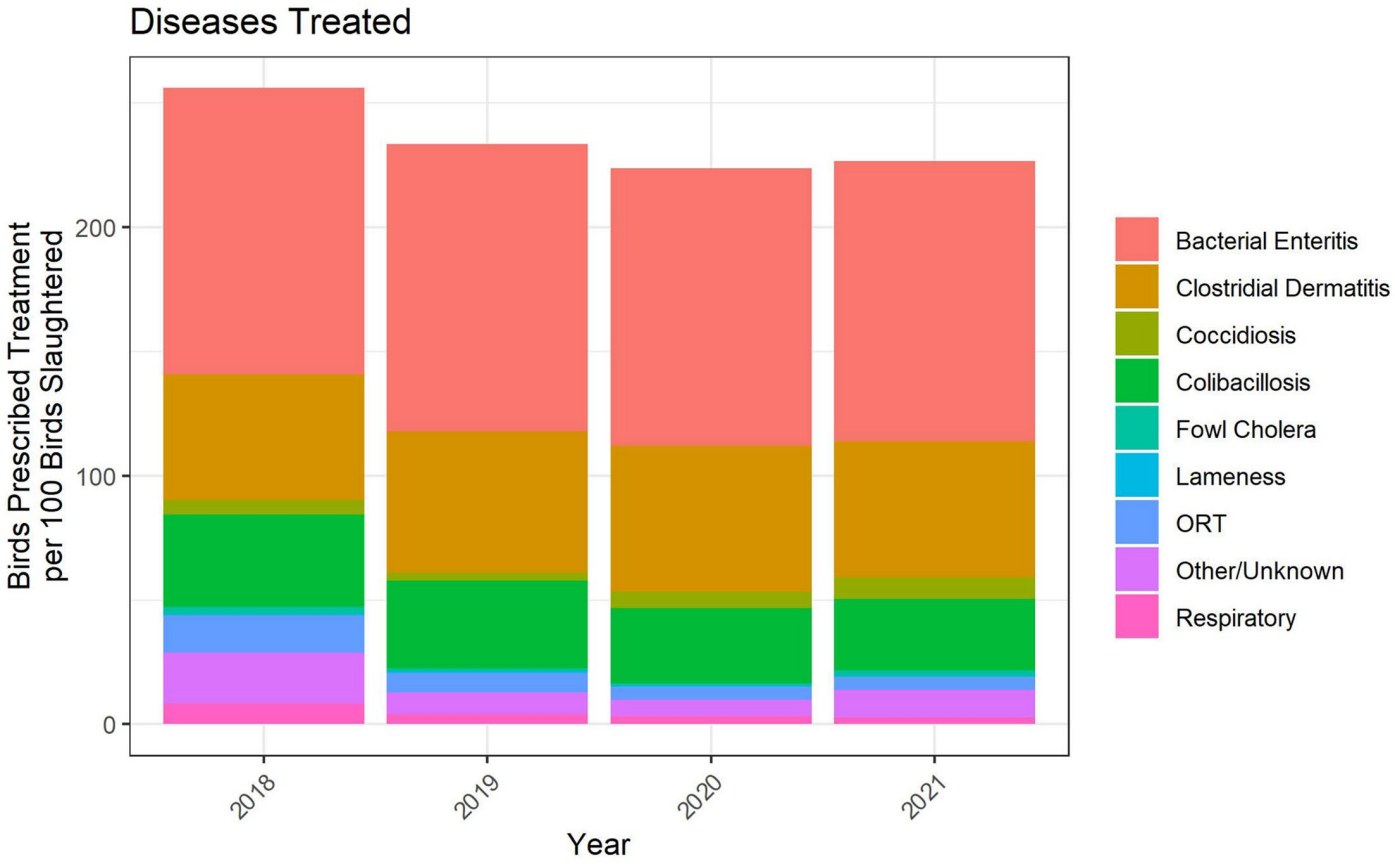
Disease indications treated with water-soluble antimicrobial administrations, 2018–2021. The figures depict the number of prescriptions for each disease indication per 100 birds slaughtered.

Flock-level treatment records for antimicrobials administered *via* the water represented between 60 and 70% of the turkeys in the 2018–2021 dataset. The indications for treatment were categorized into nine disease classifications, one of which was Other/Unknown (Figure 8). There are no standardized disease classifications within the U.S. turkey industry. We therefore categorized the recorded disease indications with input from participating veterinarians. Bacterial enteritis, clostridial dermatitis (CD) and colibacillosis were the three main disease classifications for which water-soluble antimicrobials were used when assessed with the metric of birds prescribed treatment per 100 birds slaughtered. The number of birds prescribed treatment per 100 birds slaughtered was fairly stable over the period 2018–2021.

**Figure 9:**
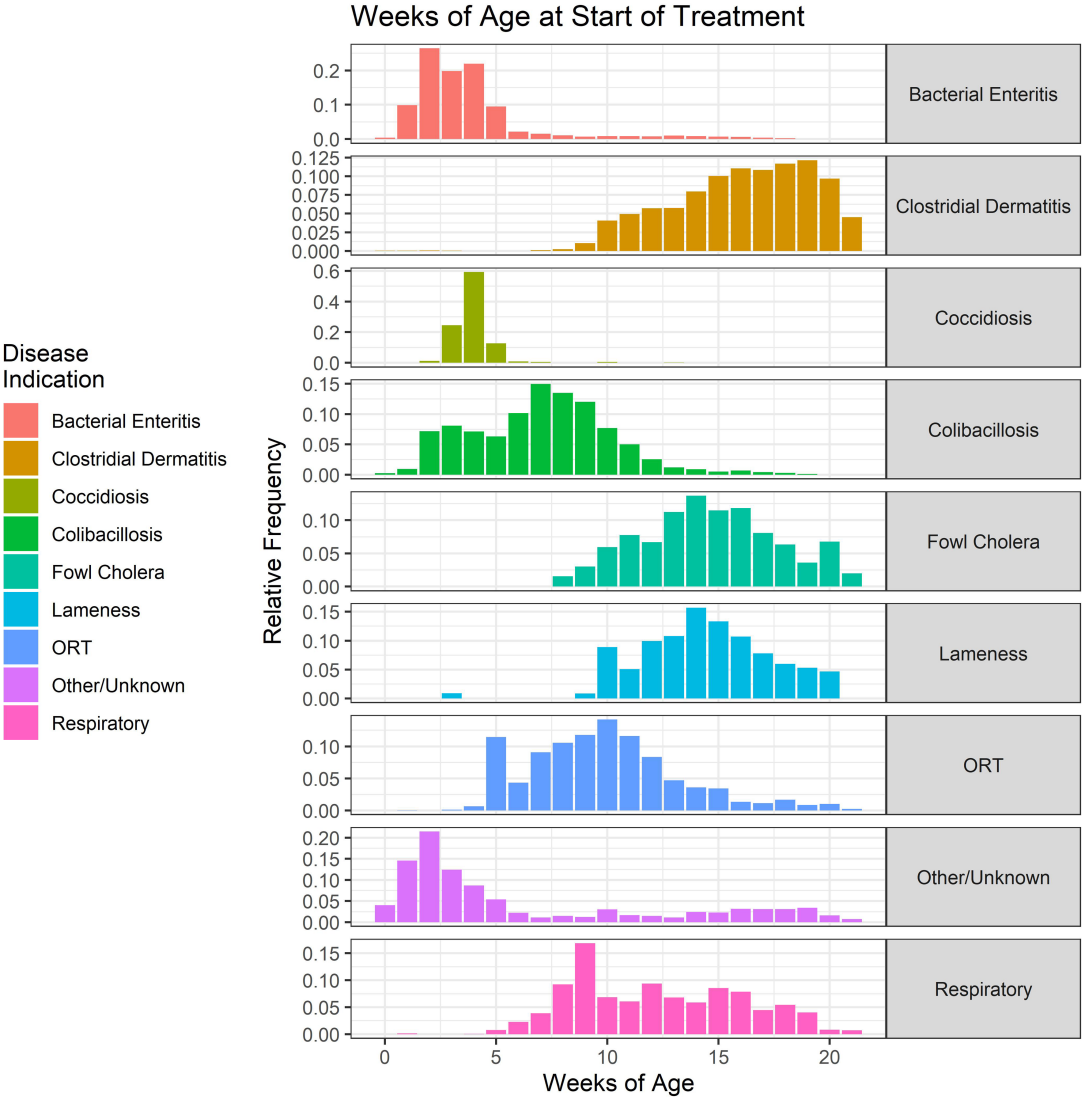
Age (in weeks) of the start of antimicrobial administrations in the water of turkeys by disease indication, 2018–2021. Each disease indication panel depicts the relative frequency of antimicrobial starting ages (in weeks) for the respective disease.

The age of onset of treatment for the nine most commonly treated disease classifications over the 2018–2021 period is shown in Figure 9. Bacterial enteritis predominantly affects the young turkey, with most treatments beginning between 2 and 4 weeks of age. Conversely, CD predominantly affects the older turkey, with most treatments beginning between 14 and 20 weeks of age. Colibacillosis, which represents a number of different disease conditions caused by *E. coli* and other Gram-negative bacteria, has a wide distribution of onset ages.

**Figure 10:**
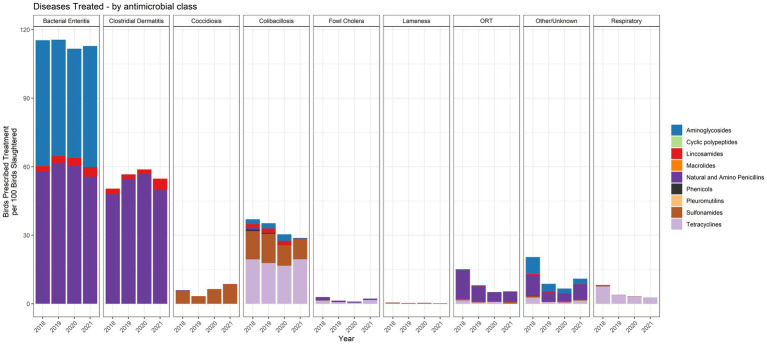
Number of water-soluble prescriptions for each antimicrobial class and disease indication, 2018–2021. The figures depict the number of prescriptions per 100 birds slaughtered.

The specific antimicrobial classes prescribed for each of the disease classifications over the 2018–2021 period are shown in Figure 10. The data are presented with the metric of birds prescribed treatment per 100 birds slaughtered. Bacterial enteritis was the disease classification with the most prescriptions per 100 birds slaughtered. This was due, in large part, to the frequent use of multiple antimicrobials prescribed at the same time, resulting in two prescriptions per bird in the affected flock. Common antimicrobial combinations used for bacterial enteritis included penicillin/gentamicin and penicillin/neomycin, as shown by the stacked columns for penicillins and aminoglycosides. Most CD treatments used penicillin, mainly as penicillin G potassium. Illnesses caused by *E. coli* were primarily treated with tetracyclines and sulfonamides.

**Figure 11:**
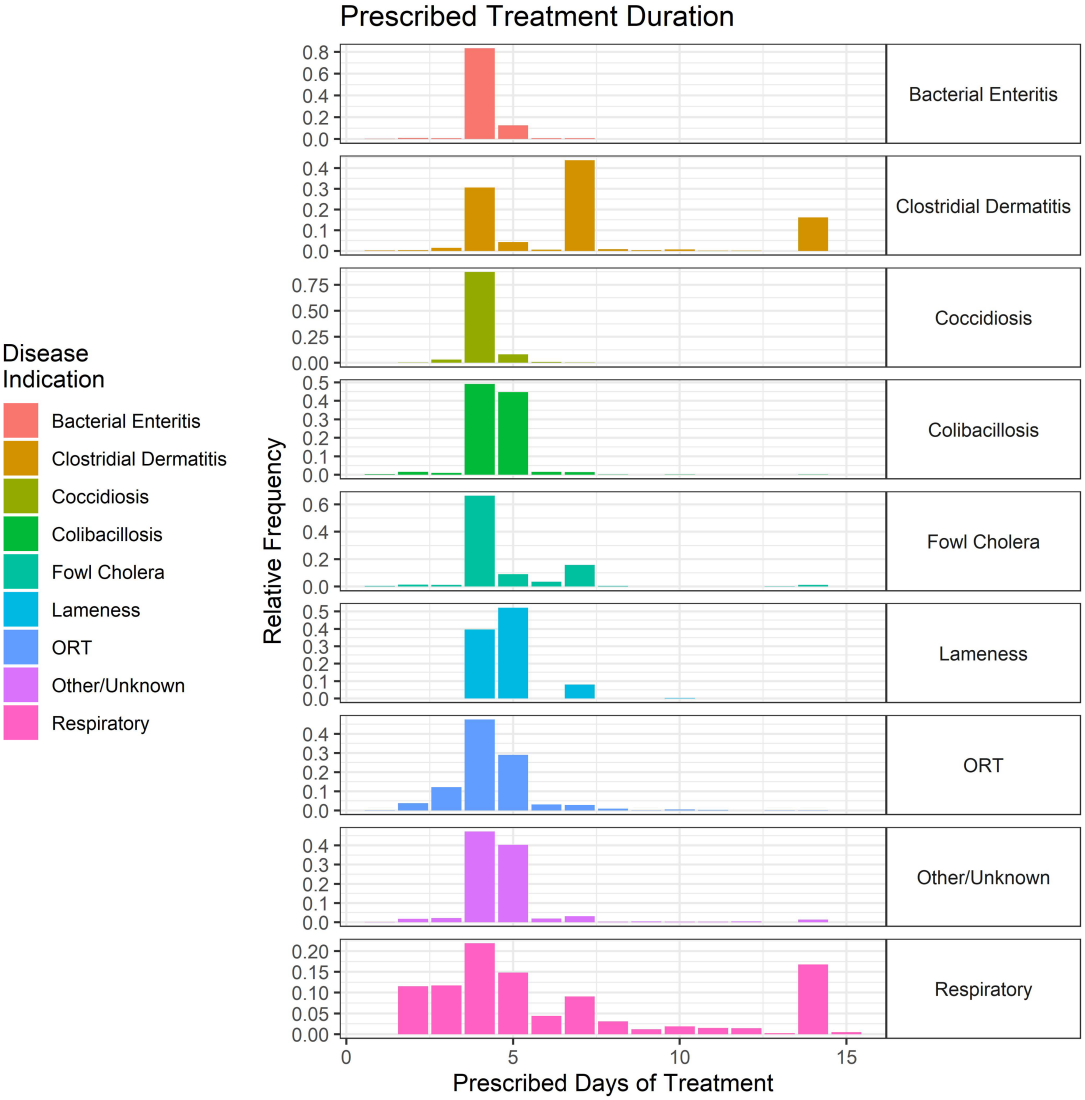
Duration (in days) of prescribed antimicrobial administrations in the water of turkeys by disease indication, 2018–2021. Each disease indication panel depicts the relative frequency of antimicrobial prescription durations (in days) for the respective disease.

The duration of treatment for these key disease classifications was variable but typically ranged from 4 to 7 days, regardless of disease (Figure 11). Some of the prescriptions for the clostridial and respiratory diseases were 14 days in duration (Figure 11). The data are shown as the relative frequency of prescription durations and are a composite of the treatment records from 2018 to 2021. Although the scale cuts off at 15 days, there were four total prescriptions in the dataset that were for longer durations (17 to 20 days), mainly for CD. Occasionally, flocks affected by CD often needed an additional prescription beyond the initial duration; due to the lack of farm identifying information in the dataset, we were unable to link these repeat prescriptions. Consequently, the number of prescriptions per 100 birds slaughtered (Figure 8) would be counting these as independent prescriptions even though they are linked to the same diseased flock and are part of the same regimen.

**Figure 12:**
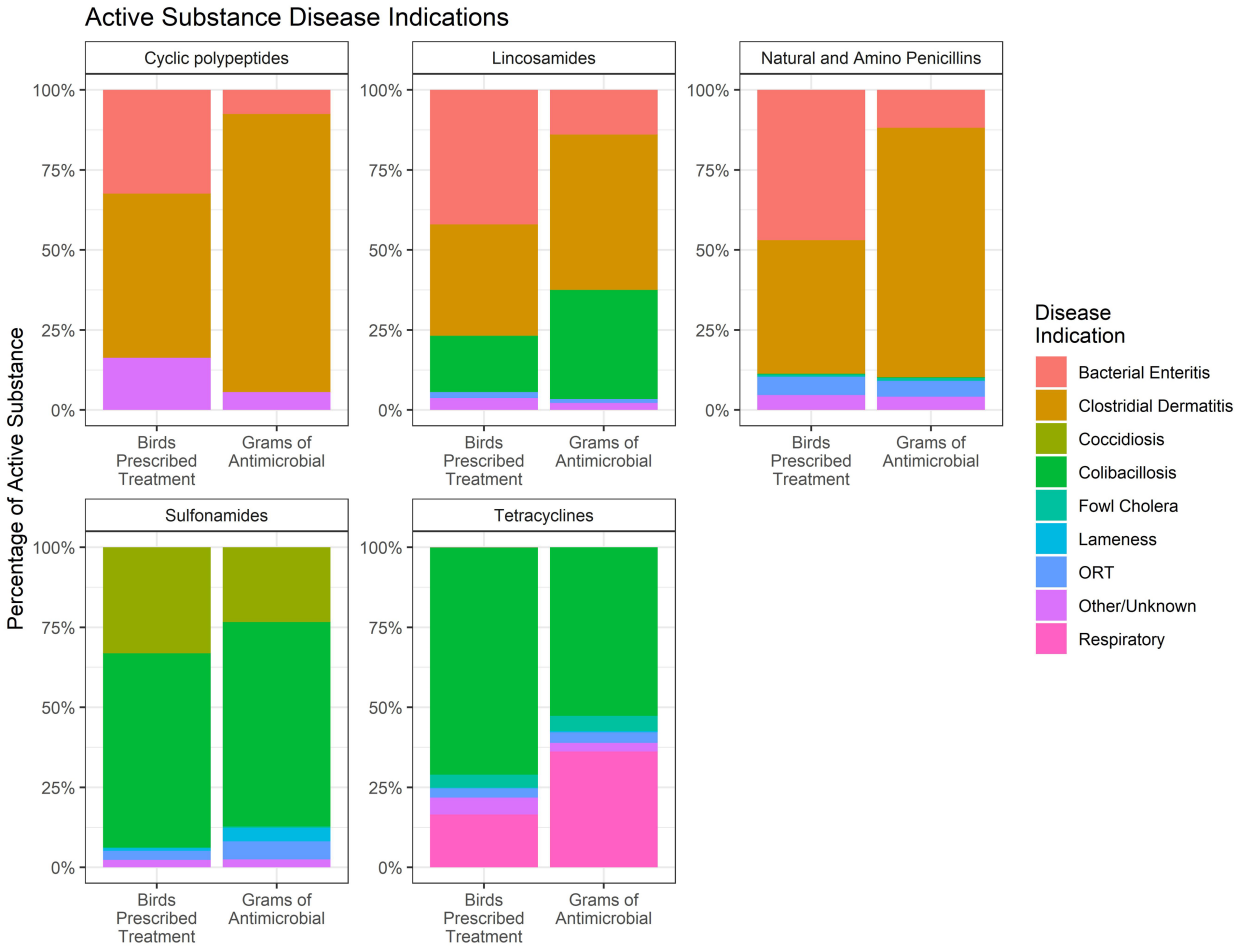
Percentage of antimicrobial use in the water of turkeys by disease indication, 2018–2021. The figures only include data for those birds that received the given antimicrobial. The figures depict the percentage of birds receiving therapy with the given antimicrobial (Birds Prescribed Treatment) and the percentage of total grams of the antimicrobial used by disease indication (Grams of Antimicrobial).

Figure 12 shows the percentage of each water-soluble antimicrobial that was administered for each disease indication. Data are presented as the percentage of birds prescribed treatment and the total grams of each antimicrobial administered for each disease indication. Penicillin and lincomycin were both used for the treatment of CD and bacterial enteritis. The figure shows that the percentage of birds receiving penicillin or lincomycin for these two diseases was about equal. However, the use of penicillin and lincomycin, by weight, was greater for CD than for bacterial enteritis. The disparity between the two measures is due to the fact that CD affects the older birds whereas bacterial enteritis affects the younger birds, and therefore, the proportion of use measured by weight (grams) is greater than the proportion of use measured by number of birds for CD.

## 4. Discussion

This study presents antimicrobial use data from U.S. commercial turkey production during the period 2013 to 2021. Companies voluntarily participated in this effort, and participation rates remained fairly consistent over the study period, with approximately 71.4% of 2021 U.S. turkey production being included in this analysis (Table 1). We were able to obtain granular treatment records from 60 to 70% of the turkeys in the 2018–2021 dataset.

As reported in our previous publication (6), antimicrobial use in the U.S. turkey sector had been declining between 2013 and 2017. Potential reasons for these declines included the policy changes made by the U.S. FDA, as previously described. However, external factors may have also influenced the patterns of use of specific antimicrobials in this dataset. For example, there has been a documented shortage of penicillin, beginning during the COVID pandemic (18). With difficulties obtaining penicillin, turkey veterinarians were forced to use a different antimicrobial for diseases such as CD or to forgo treatment of diseased flocks altogether. Based on conversations with veterinarians and based on the data, switching to lincomycin or oxytetracycline was often the decision. Finally, some companies increased their production of birds raised without antimicrobials. Current estimates for the percentage of U.S. turkey production being raised without antimicrobials range from 15% to 25% (19).

Aside from patterns of AMU being influenced by antimicrobial availability, there were other possible limitations to this effort. First, although the dataset captured the majority of U.S. turkey production, the effort only targeted the major turkey companies. No attempts were made to determine the characteristics of turkey production companies not included in this study. Another limitation of the current study is the absence of information regarding therapeutic outcomes following antimicrobial administration.

A major limitation of this data collection effort is the inability to link prescriptions to specific flocks due to the anonymization of the data. This limitation affects the analysis of the granular data in a couple of ways. First, for treatment of bacterial enteritis, multiple antimicrobials were frequently prescribed at the same time (Figure 10), resulting in two prescriptions per bird in the affected flock. This is often called regimen stacking, as each prescription represents a regimen. Further, there are times when the birds drink more water than anticipated and thus additional antimicrobial must be sent to the farm to finish the course of treatment. This will appear as a new prescription for a very short duration. Because these antimicrobials are being used to treat the same disease occurrence in a single flock, there would be four prescriptions representing two antimicrobial regimens in this example. Because the prescriptions cannot be linked to a single flock due to the anonymization of the data, we are unable to calculate precisely the number of birds treated and cannot calculate an accurate number of regimens; we would be double counting all birds in the flock that received multiple antimicrobials simultaneously for bacterial enteritis. Next, veterinarians in this project were often writing prescriptions for a shorter duration in the later years of this collection effort, especially for the clostridial diseases. If the disease continued beyond the initial prescription, the veterinarian would write an additional prescription. Again, this represents a treatment of the same disease in the same flock, and these multiple prescriptions should be recorded as a single regimen. Due to the anonymization of the data, these repeated prescriptions cannot be linked to a specific flock. Going forward, we are working on a system for creating anonymized flock identifiers that would allow us to link the prescriptions written for a given flock. Finally, the records that we receive are veterinary prescriptions and do not necessarily represent actual treatment, although it would be a very rare occurrence (according to the veterinarians in this project) for an antimicrobial in a prescription not to be used.

There were several diseases for which antimicrobials were most often used by the participating companies. First, diseases linked to *Clostridium* spp. remain an important cause of morbidity and mortality in turkey production. Clostridial dermatitis, also known as cellulitis or gangrenous dermatitis, is primarily caused by *Clostridium septicum* but also by *Clostridium perfringens* (20). Clostridial dermatitis ranked fifth and third among the disease-related issues that turkey production veterinarians faced in 2018 and 2019, respectively (21). Necrotic enteritis, caused principally by *Clostridium perfringens*, can also affect turkey flocks but was grouped into the bacterial enteritis category.

Bacterial enteritis, also referred to as dysbacteriosis or poult enteritis, (22) occurs predominately in young turkeys. It is often seen secondary to enteric pathogens including viral or protozoal diseases. Direct damage from these primary pathogens to the intestines or changes to feed consumption practices as a result of a primary disease can result in the overgrowth of certain enteric bacteria (dysbacteriosis). Antimicrobial therapy is used in affected flocks to allow them to restore normal intestinal flora. In a study by the EFFORT consortium on 60 conventional turkey farms in France, Germany and Spain (20 farms/country), poult enteritis was stated as the most important disease needing antimicrobial treatment, and consequently, the majority of treatments in the followed flocks occurred between 1 and 8 weeks of age (23). Based on the number of prescriptions written for bacterial enteritis and the frequent use of multiple antimicrobials in a single affected flock as reported in our study, bacterial enteritis in poults should be a focus of future disease prevention research in order to reduce the need for antimicrobial therapy.

Colibacillosis, which broadly refers to any localized or systemic infection caused entirely or partly by avian pathogenic *Escherichia coli* (APEC) (24), remains an important disease category in turkey production. This disease category includes conditions such as septicemia, respiratory disease, peritonitis, salpingitis, omphalitis/yolk sac infection, enteritis and others. This is evidenced in the wide range of ages of treatment for colibacillosis in the current dataset (Figure 9). Antimicrobials used in the hatchery are primarily targeted to prevent disease and to decrease mortality associated with *E. coli*, such as omphalitis/yolk sac infection in the first week of life. Colibacillosis ranked second among the disease-related issues that turkey production veterinarians faced in 2018 and 2019 (21).

Finally, the bacterium *Ornithobacterium rhinotracheale* causes a highly contagious respiratory disease, commonly referred to as ORT. Turkey veterinarians ranked ORT as the third and fourth most important disease-related issues that turkey production veterinarians faced in 2018 and 2019, respectively (21). This disease was treated with several antimicrobial classes, including penicillins, lincosamides, sulfonamides and tetracyclines. In the UK antimicrobial report, it was noted that there was an increase in antimicrobial use in turkeys between 2020 and 2021, in part due to an increased incidence of ORT and subsequent treatment to control disease (25).

Antimicrobials used in the day-old poults at the hatchery declined considerably from 2016 to 2019 but then increased again in 2020 and 2021. In discussions with the participating veterinarians, there are multiple explanations for this. First, some of the turkey companies in the U.S. experienced severe morbidity and mortality in the young poults due to a highly virulent strain of *E. coli*. Selectively adding a hatchery antimicrobial to some of the placed poults helped manage this situation. Second, some of the veterinarians reported challenges with some of the breeder flocks, and this was likely due to industrywide effects of the COVID pandemic on typical management. For example, commercial turkeys, which are typically slaughtered by 20 weeks of age, were often staying on-farm until 22 or 23 weeks of age. There were upstream challenges in the breeder flocks as well. Poults placed from some the breeder flocks were noted to have higher than normal mortality, and consequently, hatchery antimicrobials were selectively used in poults derived from these breeders.

The primary goal of AMS programs should not be the reduction of the total amounts of antimicrobial used. As stated in the 2017 DANMAP report, “a few disease outbreaks in some farms can markedly affect and cause considerable fluctuations in the national statistics on antimicrobial usage. This was the case in late 2014 and throughout 2015” (26). The 2020 DANMAP report stated that AMU in poultry increased substantially from 2019 to 2020 due to increases in infections requiring treatment (27). Specifically, the report states that there were several *E. coli* outbreaks in older birds as well as overall increases in respiratory disease and enteritis that resulted in increased usage of tetracyclines and macrolides, respectively (27). According to the UK report for 2021, the use of penicillins in turkeys increased every year between 2017 and 2020 and lincomycin use increased between 2019 and 2021 (25). As stated previously, the use of antimicrobials in UK turkeys for the treatment of ORT increased between 2020 and 2021. The incidence of disease dictates antimicrobial use patterns, assuming that antimicrobials are being used when necessary. Changes in the amount of antimicrobial used or sold do not necessarily reflect good or bad antimicrobial stewardship. To understand the reasons for these changes, it is necessary to consider the context of these changes, for example the incidence of disease that necessitates the use of antimicrobials.

Collecting AMU data at the national level can be a challenging endeavor, especially if the data collection is meant to be representative of national production. Consequently, many national programs are of limited sampling. Canada utilizes a cross-sectional sampling; the system collected data on 98 total turkey flocks nationally in 2019, representing approximately 687,360 total turkeys (28). The approach this program in Canada uses is to select sentinel flocks from several provinces, with the number flocks proportional to the number of quota-holding producers; however, it is unclear how representative this sampling is to annual national production or antimicrobial use. A study published by the EFFORT consortium focused on antimicrobial use in 60 conventional turkey farms in France, Germany and Spain (20 farms/country) (23). The authors acknowledge that these farms were not representative of national production. Even for a national system such as DANMAP in Denmark (29), the data in the report are primarily antimicrobial sales; the usage data for poultry are not divided by species and provide no information regarding indication for use or any specific antimicrobial regimen data. In general, reports from these national programs use various antimicrobial metrics, but details concerning which diseases were being treated, ages of animals at time of treatment and other variable that might help inform stewardship are typically not included.

We have established a nationally representative on-farm system for collecting AMU data from commercial turkey production in the U.S. This effort included the collection of granular flock-level data for the water-soluble prescriptions. While our prior publication reported antimicrobial totals used in turkey production over time (6), this second phase has detailed information regarding disease indications, age of birds at time of therapy, and duration of treatment. This additional information can help the industry focus on those diseases that are necessitating the majority of antimicrobial use, especially the medically important antimicrobials. For the 2018–2021 dataset, bacterial enteritis in the poults and clostridial dermatitis in the older turkeys are the two diseases that deserve increased focus for disease prevention. This project highlights a successful public-private partnership to enable collection and protection of sensitive flock-level data from an extremely large industry while releasing deidentified and aggregated information regarding the details of AMU on U.S. turkey farms over time.

## Data availability statement

The original contributions presented in the study are included in the article/Supplementary material, further inquiries can be directed to the corresponding author.

## Ethics statement

Ethical review and approval was not required for the study on animals in accordance with the local legislation and institutional requirements.

## Author contributions

RS conceived of the project, recruited the companies, coordinated data acquisition, and wrote the manuscript drafts. RS, NS, and IR conducted the data analysis. NS and MA contributed to the paper content. NS, MA, and IR commented on manuscript drafts. All authors contributed to the article and approved the submitted version.

## Funding

Funding for this project was made possible, in part, by the U.S. Food and Drug Administration through grant U01FD005878 and support from the U.S. Poultry & Egg Association (USPOULTRY).

## Conflict of interest

RS and IR were employed by Mindwalk Consulting Group, LLC. NS was employed by Livestock Veterinary Resources, LLC.

The remaining author declares that the research was conducted in the absence of any commercial or financial relationships that could be construed as a potential conflict of interest.

## Publisher’s note

All claims expressed in this article are solely those of the authors and do not necessarily represent those of their affiliated organizations, or those of the publisher, the editors and the reviewers. Any product that may be evaluated in this article, or claim that may be made by its manufacturer, is not guaranteed or endorsed by the publisher.
