# Supplementary material for: On-farm antimicrobial usage in commercial turkey production in the United States, 2013–2021
Source: Front Vet Sci. 2023 Jun 5;10:1158943. doi: 10.3389/fvets.2023.1158943 (PMC10277642; doi:10.3389/fvets.2023.1158943)
Supplement: Supplementary file 2 [file Data_Sheet_1.PDF]

Table S1. Antimicrobials that are Medically Important (MI) and that were used in turkey production in the U.S. in 2021, categorized by route of administration. Antimicrobial drugs that were used within each class are shown as well as classification of importance per Appendix A of FDA's GFI #152 (U.S. Food and Drug Administration, 2003).

Route of Administration	Drug Class	Classification	Active Ingredient
**Injectable**			
	Aminoglycosides	Highly Important	Gentamicin
	3rd Generation Cephalosporins	Critically Important	Ceftiofur
	Natural penicillins	Highly Important	Penicillin G
**Feed**			
	Tetracyclines	Highly Important	Chlortetracycline<br>Oxytetracycline
**Water**			
	Aminoglycosides	Highly Important	Gentamicin<br>Neomycin<br>Spectinomycin
	Amphenicols	Highly Important	Florfenicol
	Lincosamides	Highly Important	Lincomycin
	Diaminopyrimidines	Critically Important	Trimethoprim
	Macrolides	Critically Important	Erythromycin<br>Tylosin
	Natural penicillins	Highly Important	Penicillin G
	Sulfonamides	Critically Important	Sulfadimethoxine<br>Sulfamerazine<br>Sulfamethazine<br>Sulfaquinoxaline<br>Sulfadiazine
	Tetracyclines	Highly Important	Chlortetracycline<br>Oxytetracycline<br>Tetracycline

Table S2. Antimicrobials that are Not Medically Important (NMI) and that were used in turkey production in the U.S. in 2021, categorized by route of administration. Antimicrobial drugs that were used within each class are shown.

Route of Administration	Drug Class	Active Ingredient
**Feed**		
	Glycolipids	Bambermycins
	Ionophores	Lasalocid
		Monensin
	Polypeptides	Bacitracin
**Water**		
	Polypeptides	Bacitracin
	Pleuromutilins	Tiamulin

REFERENCES

U.S. Food and Drug Administration (2013). *Guidance for Industry #213: New Animal Drugs and New Animal Drug Combination Products Administered in or on Medicated Feed or Drinking Water of Food-Producing Animals: Recommendations for Drug Sponsors for Voluntarily Aligning Product Use Conditions with GFI #209*. Retrieved from https://www.fda.gov/regulatory-information/search-fda-guidance-documents/cvm-gfi-213-new-animal-drugs-and-new-animal-drug-combination-products-administered-or-medicated-feed. Last accessed December 23, 2022.
